# An Emerging Role for Radiation Oncology in Precision Oncology

**DOI:** 10.1016/j.ebiom.2016.03.003

**Published:** 2016-03-04

**Authors:** Sharad Goyal, Bruce G. Haffty

**Affiliations:** Department of Radiation Oncology, Rutgers Cancer Institute of New Jersey and Rutgers Robert Wood Johnson Medical School, New Brunswick, NJ, USA

Precision medicine is rapidly evolving in the management of cancer, in an effort to deliver the right therapy to the right patient at the right time. The use of novel molecular or genetic signatures in decision making regarding local–regional management is in its infancy.

In their recent work published in *EBioMedicine*, Cheng and colleagues developed an 18-gene classifier capable of stratifying breast cancer patients with 0–3 positive lymph nodes (N0–1) after mastectomy into low and high risk groups to better determine the need for post-mastectomy radiation therapy (PMRT)(Cheng et al., 2016). Through their work, they found that patients, who were defined by the 18-gene classifier as high risk, had significantly reduced rates of 5-year loco-regional recurrence, distant metastasis-free survival, and overall survival, regardless of lymph node status and breast cancer subtype based on estrogen receptor, progesterone receptor, and human epidermal growth factor receptor 2/neu status.

The importance of this study lies in the fact that the gene expression profile developed by this group to support the decision for PMRT or not, which is in many ways similar to the Oncotype Dx 21-gene panel, which helps support the decision for adjuvant chemotherapy. This example of gene expression profiling is exactly what is necessary to move the field of radiation oncology forward towards precision medicine (PM). PM should not only integrate genomic data, but also relevant clinical, pathologic, and environmental parameters significant to the patient (Servant et al., 2014). The ability to improve outcomes in breast cancer using radiation therapy should be focused on incorporating novel genomic- or biology-based biomarkers, in addition to existing clinical and pathologic features, in the treatment of patients.

Radiation therapy is a cornerstone of modern multidisciplinary breast cancer treatment. In the recent past, strategies to improve the outcome of patients undergoing radiation therapy have centered on: 1. Advanced imaging of tumor morphology and function for radiotherapy planning (e.g. functional imaging); 2. Improving the therapeutic ratio through the precision of radiotherapy delivery (e.g. computational algorithms, IMRT, IGRT, SBRT); and 3. Incorporation of novel radiosensitizing and molecular targeting agents with radiation therapy to reduce mechanisms of radiation resistance (e.g. epithelial growth factor receptor inhibitors and RT in head and neck cancer). Despite these achievements, determining the benefit of radiation therapy based on the molecular profile of a tumor remains largely unresolved across the spectrum of cancers. It is critical for radiation oncologists to systematically evaluate tailored treatment strategies for various malignancies based on the individual genetic profile of cancer (Collins and Varmus, 2015).

In reporting these results, the authors presented a shifting paradigm in breast radiation oncology, namely using gene expression profiles to identify patients likely to benefit from PMRT in lieu of standard clinical and pathologic features (Cheng et al., 2016). Weighing the potential improvement in overall survival with PMRT against the potential long-term toxicities associated with PMRT, namely potential cardiopulmonary toxicity and secondary malignancies, we are hopeful that in the future, gene expression profiles will help spare low-risk patients unlikely to benefit from PMRT and truly identify those patients at high risk of recurrence that would benefit from PMRT.

Prospective validation of this gene expression profile in a larger cohort of patients will be necessary to determine its prognostic and predictive impact in patients undergoing mastectomy. In addition, the cost-effectiveness of gene expression profiles versus that of standardized guidelines for patients who would benefit from PMRT needs to be estimated. Studies involving molecular profiling need to be developed to help usher the field of radiation oncology into the era of precision medicine in the 21st century.

## Disclosure

The authors declared no conflicts of interest.

## References

[bb0005] Cheng S.H., Horng C., Huang T. (2016). An eighteen-gene classifier predicts local/regional recurrence in post-mastectomy breast cancer patients. EBioMedicine.

[bb0010] Collins F.S., Varmus H. (2015). A new initiative on precision medicine. N. Engl. J. Med..

[bb0015] Servant N., Romejon J., Gestraud P. (2014). Bioinformatics for precision medicine in oncology: principles and application to the SHIVA clinical trial. Front. Genet..

